# Functional Analysis of the *Scarlet* Gene in the Cricket *Gryllus bimaculatus*

**DOI:** 10.3390/insects17010033

**Published:** 2025-12-25

**Authors:** Li-Fen Zeng, Yun Bai, Long Chen, Xin-Kun Yang, Jin-Li Xu, Zhu-Qing He, Kai Li

**Affiliations:** Laboratory of Entomology, School of Life Science, East China Normal University, Shanghai 201100, China; 51271300001@stu.ecnu.edu.cn (L.-F.Z.); 52261300063@stu.ecnu.edu.cn (Y.B.); cl0111@163.com (L.C.); yxkmimi@163.com (X.-K.Y.); 10231910061@stu.ecnu.edu.cn (J.-L.X.)

**Keywords:** *Gryllus bimaculatus*, *scarlet* gene, compound eye pigmentation, CRISPR/Cas9, ABC transporter

## Abstract

The *scarlet* gene encodes an ATP-binding cassette transporter involved in eye pigmentation across various insect species. In this study, we functionally characterized the *scarlet* homolog (*Gbst*) in the cricket *Gryllus bimaculatus*, a hemimetabolous model organism. Clustered regularly interspaced short palindromic repeats/CRISPR-associated nuclease 9-mediated knockout of *Gbst* generated a stable yellow-eyed mutant line (*Gbst*^−/−^) with changed pigmentation evident from embryogenesis through adulthood. Quantitative real-time PCR analysis showed that *scarlet* expression was extremely low in *Gbst*^−/−^, and the transcript levels of *white* and *brown* were also reduced. Histological sections of the compound eyes showed that both WT and *Gbst*^−/−^ mutant possessed complete and well-defined ommatidial structures, indicating that the *scarlet* gene does not affect compound eye structure. In addition, reproduction tests showed that knockout of the *Gbst* gene did not affect egg production or embryonic viability. These findings demonstrate that *Gbst* is a key factor involved in eye pigmentation in *G*. *bimaculatus*, and has potential for application as a visual transgenic marker gene.

## Simple Summary

Eye-color genes are widely used to study insect development and genetics. In this work, we focused on the *scarlet* gene in the cricket *Gryllus bimaculatus*. By applying CRISPR/Cas9 gene editing, we produced a stable yellow-eyed mutant line, in which the pigmentation defect was visible from the embryonic stage to adulthood. Despite the altered eye color, the microscopic structure of the compound eyes and the reproductive capacity of the *Gbst*^−/−^ knockout strain were unaffected. These results show that this gene plays a specific role in eye pigmentation but not in eye development or fertility. Therefore, this gene can be considered a useful visible marker for genetic manipulation in crickets.

## 1. Introduction

Eye-color mutants in insects have served as valuable models in various biological research domains [1,2,3,4,5]. Early investigations of such mutants in *Drosophila melanogaster* provided foundational evidence for the chromosomal theory of inheritance, notably facilitating the first assignment of a specific gene to a defined locus on a sex chromosome [6,7]. Research in this area has predominantly focused on holometabolous insect taxa, including Diptera [8,9], Coleoptera [10], Hymenoptera [11], and Lepidoptera [12,13]. Among hemimetabolous groups, the order Hemiptera includes the most extensively characterized eye-color mutants [14,15,16,17]. The genes responsible for eye pigmentation typically encode amino acid sequences involved in pigment synthesis and transport, and also participate in a variety of physiological processes unrelated to vision, thereby exhibiting pleiotropic functions [2,10,18,19,20]. These characteristics have made eye-color mutants valuable tools in diverse biological studies and potential visible markers for transgenic research in multiple insect species [10,21].

The pigmentation of insect compound eyes is primarily governed by the biochemical properties and distribution of distinct classes of pigments [4]. Among these, ommochromes and pteridines represent the two major pigment types essential for eye coloration [4,22,23,24,25]. Ommochromes are metabolic derivatives of the tryptophan catabolic pathway, whereas pteridines are biosynthesized from guanosine triphosphate (GTP) [4]. Disruptions in the activity of enzymes or transporters involved in these pigment biosynthetic pathways can lead to abnormal pigment accumulation or depletion, thereby altering the characteristic wild type (WT) eye colour phenotype [10,11,26]. Mutations affecting eye pigmentation in insects can be classified into four primary categories, depending on the specific biosynthetic or cellular pathways involved: (1) xanthommatin synthesis, (2) pteridine synthesis, (3) transmembrane transport of pigments or their precursors, and (4) the biogenesis of pigment granules [22,27,28,29]. In *D*. *melanogaster*, a number of genes associated with these processes have been well characterized [18,30]. The transport of pigments and their precursors across intracellular membranes relies predominantly on three ATP-binding cassette (ABC) transporters genes: *white*, *scarlet*, and *brown* [29,31].

The gene *scarlet*, first well-characterized in *D*. *melanogaster*, exhibits nucleotide sequence homology with the *white* gene, suggesting that both function as membrane-associated ABC transporters involved in the translocation of pigment precursors [32]. Accordingly, *scarlet* is essential for normal eye pigmentation and contributes critically to the formation of typical eye coloration in diverse insect species [32,33,34,35,36,37]. The *scarlet* gene in *D*. *melanogaster* (Diptera) plays a critical role in the intracellular transport of ommochrome precursors, and mutations in this gene disrupt normal pigment deposition, resulting in abnormal eye colour phenotypes, typically characterized by dark red eyes with mottled or spotted patterns in *scarlet* mutants [32]. In *Chrysodeixis includens* (Lepidoptera), Clustered regularly interspaced short palindromic repeats (CRISPR)/CRISPR-associated nuclease 9 (Cas9)-mediated knockout of the *scarlet* gene leads to mutant adults with light greenish or yellowish compound eyes, and no significant differences in other physiological traits compared to the WT [33]. In *Papilio xuthus* (Lepidoptera), F_0_ adults with *scarlet* gene knockout showed abnormal eyes with white-black or red-brown mosaic stripes, while F_2_ adults developed completely white eyes [34]. In *Nilaparvata lugens* (Hemiptera), RNA interference (RNAi)-mediated knockdown of the *scarlet* gene in 1st to 3rd-instar nymphs leads to a darkened eye color in adults, accompanied by significant reductions in xanthommatin and pteridine levels in the eyes [35]. In *Tribolium castaneum* (Coleoptera), RNAi knockdown of *Tcst* (the *scarlet* ortholog) induces a white-eyed phenotype in adults that persists for at least two weeks after eclosion, but does not last throughout the adult lifespan [36]. In *Harmonia axyridis* (Coleoptera), knockdown of the *scarlet* gene specifically affects eye pigmentation, resulting in white-eyed adults while leaving body coloration unaffected [37].

*Gryllus bimaculatus* (Orthoptera: Gryllidae) is a well-established model organism for the study of insect development, regeneration, behavior, neurobiology and physiology [38,39,40,41]. ABC transporter genes in Orthoptera remain relatively understudied, with only the *white* gene providing insight [42]. While genome sequencing, assembly, and annotation have been performed for the white-eyed strain of *G*. *bimaculatus* (which displays a yellowish compound-eye appearance) [41], the molecular mechanism underlying its pigmentation phenotype remains unelucidated to date. We hypothesize that this eye-color mutant is associated with the *scarlet* gene. In this study, we characterized the *scarlet* gene in *G*. *bimaculatus* (*Gbst*). CRISPR/Cas9-mediated knockout of *Gbst* was then employed to investigate its role in the eye pigment pathway. Our results demonstrate that *Gbst* regulates eye pigmentation in *G*. *bimaculatus*.

## 2. Materials and Methods

### 2.1. Insect Culture and Egg Collection

The *G*. *bimaculatus* populations used in this study were obtained by laboratory rearing [43]. Crickets were reared on a diet of fish food in a 12 h light and 12 h dark photoperiod at a constant temperature of 30 °C and a relative humidity of 60% ± 5%, housed in plastic boxes (10 cm × 6 cm × 13 cm). Three days before microinjection, five males and five females were placed in the rearing box for mating. Three days after mating, the females were then transferred to the plastic box to lay eggs.

### 2.2. Identification and Sequence Analysis of the Gbst

The putative *Gbst* gene was identified by BLASTP search of the *G. bimaculatus* genome assembly (GenBank accession: GCA_017312745.1; BioProject: PRJDB10609) using the amino acid sequence of *B. germanica* SCARLET (GenBank: PSN52318.1) as a query [41]. The genome assembly and annotations are available from the original data portal (http://gbimaculatusgenome.rc.fas.harvard.edu, accessed on 12 October 2023) and NCBI [41]. The open reading frame (ORF) and corresponding amino acid sequence of the *Gbst* gene were predicted using the Translation tool available on the ExPASy server (http://web.expasy.org/translate/, accessed on 5 November 2023). The physicochemical characteristics of the translated amino acid sequence were assessed via ProtParam, an online tool provided by ExPASy (https://web.expasy.org/protparam/, accessed on 5 November 2023). Conserved amino acid sequence domains were identified using the Conserved Domain Database (CDD) search tool hosted by NCBI (https://www.ncbi.nlm.nih.gov/Structure/cdd/wrpsb.cgi, accessed on 20 December 2023). A gene tree was constructed based on aligned sequences of three ABC transporter gene members (*scarlet*, *white*, and *brown*). The selection of these specific genes was necessary to distinguish them effectively, given the high sequence similarity within this gene family. Phylogenetic relationships were inferred through the neighbor-joining (NJ) method, and the robustness of the resulting tree topology was evaluated by bootstrap analysis with 1000 replicates. In this study, the amino acid sequences of SCARLET from *Aedes aegypti* (XP_011493242.2), *Anabrus simplex* (XP_066997954.2), *B*. *germanica* (PSN52318.1), *Diachasma alloeum* (XP_015116893.1), *D*. *melanogaster* (NP_524108.1), *Musca domestica* (XP_011295262.1), *P*. *xylostella* (XP_011564256.1), and *Tribolium castaneum* (AJD07060.1); WHITE homologues from *Acyrthosiphon pisum* (XP_001943103.2), *Amyelois transitella* (XP_013190063.1), *Bemisia tabaci* (XP_018908689.1), *Camponotus floridanus* (XP_011259217.1), *D*. *melanogaster* (NP_476787.1), *Helicoverpa armigera* (XP_021185359.1), *Ooceraea biroi* (XP_011340446.1), *Plutella xylostella* (XP_011564267.1), *T*. *castaneum* (NP_001034521.1), and *Trichogramma pretiosum* (XP_014230795.1); and BROWN from *Athalia rosae* (XP_012251836.1), *Bombyx mori* (BAN66701.1), *D*. *melanogaster* (NP_001286769.1), *H*. *armigera* (ANW09742.1), *Linepithema humile* (XP_012235630.1), *Megachile rotundata* (XP_012153544.1), *O*. *biroi* (EZA57078.1), *Papilio polytes* (XP_013149403.1), *Pieris rapae* (XP_022114946.1), *Pseudomyrmex gracilis* (XP_020287473.1), *Solenopsis invicta* (XP_011166210.1), and *T*. *castaneum* (AJD07061.1) were downloaded from the NCBI amino acid sequence database (https://www.ncbi.nlm.nih.gov/protein/, accessed on 15 May 2024).

### 2.3. In Vitro Transcription of Gbst Single-Guide RNA (sgRNA)

The sgRNA targeting *Gbst* was designed using the CRISPOR online tool (https://crispor.gi.ucsc.edu/crispor.py, accessed on 20 May 2024). Candidate target sites were evaluated for predicted on-target efficiency and minimal off-target effects. The site with the highest predicted specificity and efficiency was chosen for sgRNA synthesis. The sgRNA templates were prepared using a pair of primers (Table 1). The sgRNA was generated by in vitro transcription using the MEGAscript™ T7 kit (Thermo Fisher Scientific, Waltham, MA, USA) according to manufacturer’s instructions. The reaction conditions were as follows: 94 °C for 5 min, 35 cycles of 94 °C for 15 s, 56 °C for 15 s, and 72 °C for 15 s, followed by a final extension period of 72 °C for 10 min. As outlined in the study by Bai et al. [43], the efficiency of sgRNA synthesis and cleavage was subject to experimental validation, and the purified sgRNA were stored in aliquots at −80 °C.

### 2.4. Embryo Microinjection

Fertilized eggs were harvested within 2 h post-oviposition, arranged in grooves containing 1% agarose gel, and subjected to microinjection within 2 h of alignment using a microinjector. The injection mixtures comprised buffer, Cas9 protein and sgRNA synthesized in vitro. The concentration of Cas9 was maintained at 300 ng/μL, while that of sgRNA was set at 500 ng/μL. The injected eggs were then subjected to incubation at a temperature of 30 ± 0.5 °C, under conditions of 60% ± 5% humidity and a 12 h light and 12 h dark cycle.

### 2.5. Mutational Identification and Germline Transmission

To confirm mutagenesis at the *Gbst* locus, genomic DNA was extracted from whole individual eggs, as well as from the dissected antennae of nymphs and adults. For all samples, DNA was isolated using a commercial DNA extraction buffer (Transgen, Beijing, China) according to the manufacturer’s protocol. The DNA was then incubated with proteinase K and purified using the EasyPure^®^ Genomic DNA Kit (Transgen, Beijing, China). Subsequently, the genomic DNA was utilized as a template for amplification. PCR was performed to amplify the regions surrounding the site of the sgRNA. The product of this process was a 284 bp fragment. The amplified products were ligated into the pGEM^®^-T vector (Promega, Madison, WI, USA) and subsequently subjected to sequencing. The primers employed in this study were designated *Gbst*-F/R (Table 1). To generate a stable *Gbst* mutant line, F_0_ individuals carrying mutations were backcrossed with WT crickets to produce the F_1_ generation. Genomic DNA was isolated from the antennae of each F_1_ adult and used as a template to determine mutation types. F_1_ heterozygous individuals harboring identical mutations were crossed to produce the F_2_ generation. Homozygous *Gbst* mutant (*Gbst*^−/−^) were screened from F_2_ progeny using the same genotyping strategy as described above. Mutations in the F_2_ generation were detected by amplifying genomic regions encompassing the target sites with specific primers, followed by subcloning and sequencing of the PCR amplicons.

### 2.6. Phenotype Observation

The eye color development of *Gbst*^−/−^ mutant and WT individuals was documented at four developmental stages: 1 day before hatching, 1st instar, 5th instar, and adult. Eggs were photographed while submerged in water, whereas 1st instar, 5th instar, and adult crickets were anesthetized on ice for 5 min and imaged using a Leica M125 stereomicroscope (Leica, Wetzlar, Germany).

To observe the structure of the compound eyes, the heads of 5th instar *Gbst*^−/−^ and WT nymphs were collected and processed for Hematoxylin-eosin (HE) staining of compound eye sections. Following dissection, samples were fixed for 48 h in neutral paraformaldehyde solution (Servicebio, Wuhan, China). A sequential dehydration process ensued using graded ethanol concentrations: 75% (4 h), 85% (2 h), 90% (2 h), 95% (1 h), succeeded by two incubations in anhydrous ethanol (30 min each), and an 8-min immersion in alcohol benzene. The tissues were cleared in an ethanol–xylene mixture for 8 min, followed by two 8-min incubations in pure xylene. Thorough infiltration was then accomplished by incubating the samples in melted paraffin at 65 °C for 1 h, with the wax being completely refreshed three times. After infiltration, the samples were embedded in paraffin blocks and sectioned using a microtome. These sections were stained with hematoxylin and eosin (Servicebio, Wuhan, China). Digital images of the stained sections were acquired using a slide scanning system (3DHISTECH Ltd., Budapest, Hungary) for observation. Each treatment group included three biological replicates. The stained sections were observed under an OLYMPUS BX53 microscope (Olympus Corporation, Tokyo, Japan).

### 2.7. Quantitative Real-Time PCR (qPCR) Analysis

To investigate the transcriptional impact of *Gbst* knockout on eye pigmentation pathways, we compared the expression levels of key eye coloration genes (*scarlet*, *white*, and *brown*) between WT and *Gbst*^−/−^ mutant nymphs. Total RNA was isolated from the head tissues of 8th-instar nymphs of both WT and *Gbst*^−/−^ mutant using the TransZol Up reagent kit (Transgen, Beijing, China). First-strand cDNA was then synthesized from each RNA sample with the TransScript One-Step gDNA Removal and cDNA Synthesis SuperMix kit (Transgen, Beijing, China). qRT-PCR reactions were performed using ChamQ Universal SYBR qPCR Master Mix (Vazyme, Nanjing, China) under the following conditions: initial denaturation at 95 °C for 30 s; followed by 40 cycles of 95 °C for 10 s and 60 °C for 30 s. The relative gene expression levels were calculated using the 2^−ΔΔCt^ method, normalized to the expression of *Gbtubulin*. Three biological replications were conducted for each sample. Primers used are listed in Table 1.

### 2.8. Egg Production and Embryonic Viability Analysis

Pairs of adult male and female crickets were placed in plastic boxes (28 cm × 20 cm × 14.5 cm) for mating. After mating, the female crickets were individually placed in individual plastic boxes lined with moist paper towels for egg laying. The pairs of adult crickets were allowed to mate and lay eggs over four consecutive days (mating 12 h: oviposition 12 h). Subsequently, the number of eggs laid in each box was counted. The eggs on moist paper were incubated at 30 ± 0.5 °C and 60–65% relative humidity until hatching, after which the number of hatched nymphs was recorded. For each female cricket, the hatching rate was calculated by dividing the number of nymphs by the total number of eggs produced by that individual. Three biological replicates were analyzed for each condition.

### 2.9. Statistical Analyses

The experimental data were analyzed using Student’s *t*-test (* *p* < 0.05; ** *p* < 0.01; *** *p* < 0.001; **** *p* < 0.0001) with the software programme GraphPad Prism 9.5.1 in order to compare the results between the groups.

## 3. Results

### 3.1. Identification and Analysis of Gbst

**Figure 1:**
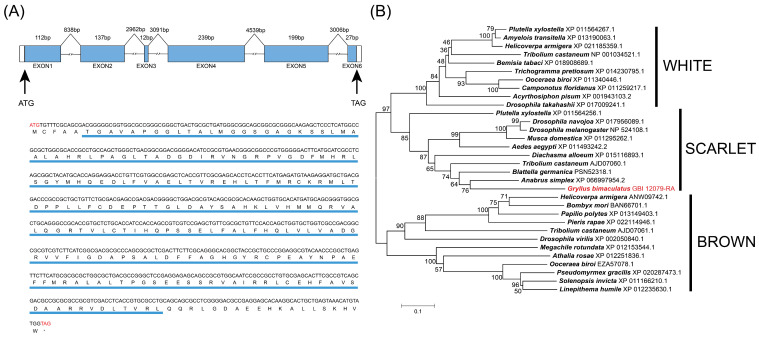
*Gbst* gene structure, amino acid sequence and phylogenetic analysis of the ABC transporter amino acid sequences. (**A**) Structural features of the *Gbst* gene, complementary DNA (cDNA), and deduced amino acid sequence. The blue boxes indicate the positions of exons, the white boxes indicate the untranslated region, and the lines represent introns. Predicted gene structure was drawn to scale. The cDNA nucleotides and deduced amino acid sequences of the *Gbst* gene correspond to each other. The start (ATG) and stop (TAG) codons in the nucleotide sequence are highlighted in red. In the corresponding protein sequence, the stop codon is represented by a ‘*’. The highly conserved region (3a01204 super family domain) of ST homologs is highlighted in blue. (**B**) Phylogenetic tree of the ABC transporter amino acid sequences among several insects. Phylogenetic tree of known ABC transporter amino acid sequences constructed by the neighbor-joining (NJ) method.

A BLASTP search of *B*. *germanica* SCARLET amino acid sequence against this reference genome sequence identifies GBI 12079-RA as the closest candidate ortholog. The coding sequence of *Gbst* is 726 bp, containing 6 exons and 5 introns (Figure 1A). The ORF encodes a 241-amino-acid sequence with a predicted molecular weight of 59,270.02 Da and an isoelectric point of 5.05. GbST amino acid sequence contains a 3a01204 super family domain (6–222), which is a conserved domain structure among SCARLET amino acid sequences (Figure 1A).

A total of 30 putative ABC transporter amino acid sequences were identified from various insect species, including 10 WHITE, 8 SCARLET, and 12 BROWN homologs. Phylogenetic analysis showed that these amino acid sequences were clearly separated into three distinct clades corresponding to the WHITE, SCARLET, and BROWN subfamilies (Figure 1B). GbST was located within the SCARLET clade and showed the closest phylogenetic relationship to *B*. *germanica* SCARLET (Figure 1B).

### 3.2. Transgenic CRISPR/Cas9-Based Mutagenesis of Gbst

**Figure 2:**
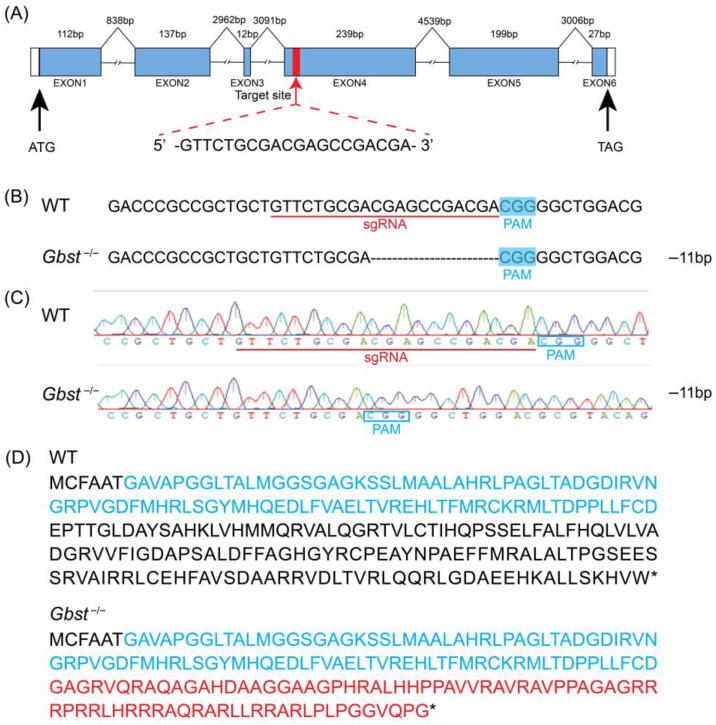
Targeted mutation of *Gbst* induced by CRISPR-Cas9. (**A**) Diagram of CRISPR/Cas9-mediated knockout system targeting to *Gbst*. The blue boxes indicate the positions of exons, the white boxes indicate the untranslated region, and the lines represent introns. Red arrow indicates single-guide RNA (sgRNA) target site, which is shown in detail. (**B**) Characterization of *Gbst*^−/−^ allele sequence deletion. Deleted bases are indicated by dashes. The blue box indicates the PAM sequence, and the sgRNA target site is marked with a red horizontal line on the WT sequence. The number of deleted bases is shown on the right side of allele (−: deletion). (**C**) Sequencing chromatograms of WT and homozygous *Gbst*^−/−^ individuals. The number of deleted bases is shown on the right side of allele (−: deletion)., resulting in a frameshift mutation. (**D**) Predicted truncated amino acid sequence products translated from WT and the *Gbst*^−/−^ alleles. 3a01204 super family domain sequences are shown in blue. Frameshifted sequence is shown in red. The “*” represents a stop codon.

The sgRNA was designed to target exon 6 of *Gbst* in order to introduce a frameshift mutation, resulting in a premature stop codon that disrupts the open reading frame and abolishes the function of the encoded protein (Figure 2A). A total of 336 eggs were subjected to microinjection, resulting in 98 successful hatchings. Among the hatched nymphs, 46 exhibited yellow eye pigmentation, while 52 displayed dark brown eyes. Of the yellow-eyed individuals, 24 successfully eclosed to adulthood. All F_1_ offspring exhibited dark brown eye pigmentation. Among the F_1_ crosses, the group produced the highest number of eggs was retained for subsequent breeding. Analysis of this group revealed a 11 bp deletion, which was predicted to change the amino acid sequence downstream of the deletion site and lead to premature termination of translation (Figure 2B–D).

### 3.3. CRISPR/Cas9 Knockout of Gbst Affects Compound Eye Pigmentation

**Figure 3:**
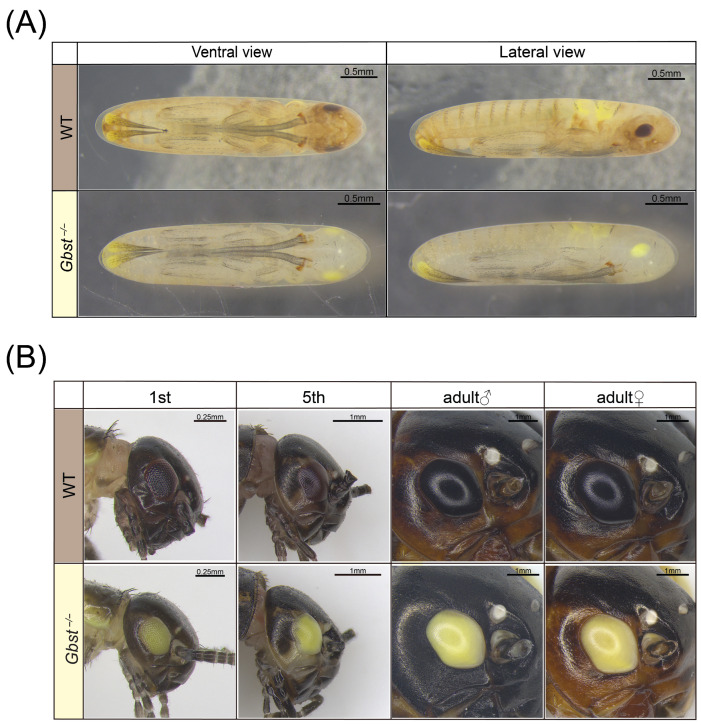
CRISPR/Cas9-mediated knockout of *Gbst* impacts eye color. (**A**) Photographs of the pre-embryonic development stage, 1 day before the egg hatching. The eye primordia of WT appear dark brown, and those of *Gbst*^−/−^ appear bright yellow. (**B**) Photographs of compound eyes of postembryonic developmental stages, including 1st, 5th, adult females and males. Some of the antennae are cut off for easy photography. The compound eyes of WT were always dark brown and *Gbst*^−/−^ showed a bright yellow color.

During embryonic development, *Gbst*^−/−^ mutant were pale yellow overall, in contrast to the brown coloration of WT individuals (Figure 3A). This color difference was most pronounced in the head and the dorsal setae on the abdomen, and was also evident in the compound eye primordium, which appeared yellow in mutant versus dark brown in the WT. In the post-embryonic developmental stage, the overall body color difference between the genotypes became less pronounced, while the distinct coloration of their compound eyes persisted. The compound eyes of WT crickets remained consistently dark brown to black, whereas those of the *Gbst*^−/−^ mutant stayed yellow throughout development (Figure 3B). In contrast, no noticeable difference in ocellus pigmentation was observed between WT and *Gbst*^−/−^ individuals (Figure 3B).

### 3.4. CRISPR/Cas9 Knockout of Gbst Does Not Affects Structure of Compound Eyes

**Figure 4:**
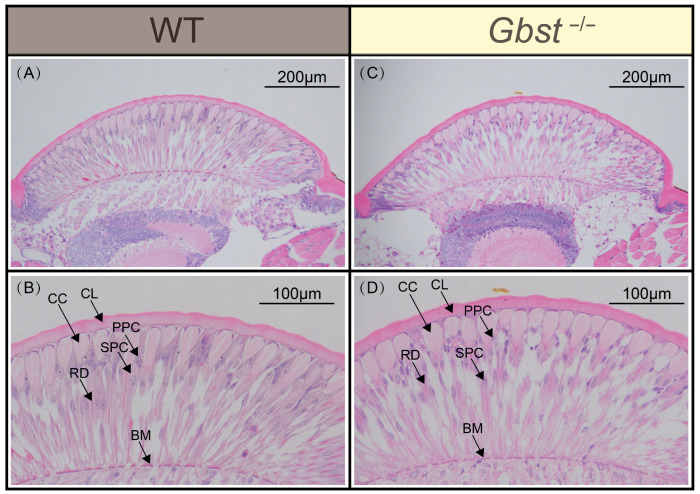
CRISPR/Cas9-mediated knockout of *Gbst* does no impacts on the structure of compound eyes. (**A**,**B**) Longitudinal section of WT compound eye. (**C**,**D**) Longitudinal section of *Gbst*^−/−^ compound eye. Corneal Lens (CL), Crystalline Cone (CC), Primary Pigment Cells (PPC), Secondary Pigment Cells (SPC), Rhabdom (RD), and Basement Membrane (BM).

Longitudinal sections of the compound eye in WT individuals revealed densely packed, fan-shaped ommatidia arranged side by side (Figure 4A). Each ommatidium functioned as an independent visual unit and exhibited a clearly defined structure, consisting, from the outer to the inner layer, of the corneal lens, crystalline cone, primary pigment cells, secondary pigment cells, rhabdom, and basement membrane (Figure 4B). The crystalline cone was surrounded by two primary pigment cells, which extended partially over the corneal lens. Similarly, in *Gbst*^−/−^ mutant, the compound eye also consisted of densely packed, fan-shaped ommatidia (Figure 4C). A detailed examination confirmed that mutant possessed the same structural components, including the corneal lens, crystalline cone, primary pigment cells, secondary pigment cells, rhabdom, and basement membrane (Figure 4D).

### 3.5. CRISPR/Cas9 Knockout of Gbst Does Not Affect Egg Production and Embryonic Viability

**Figure 5:**
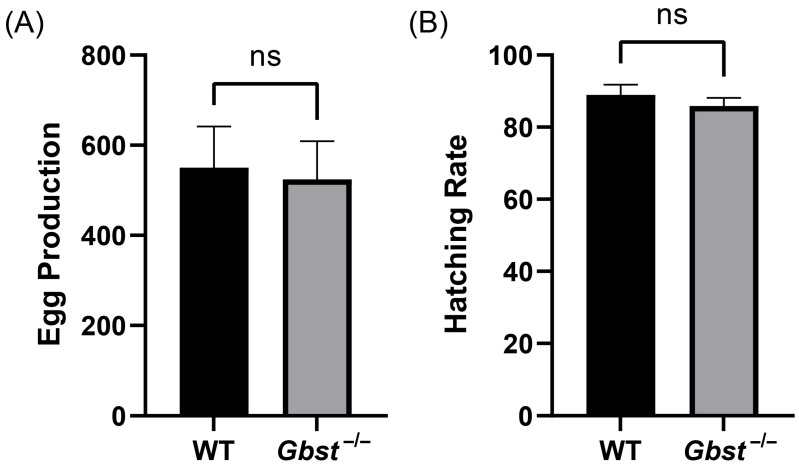
CRISPR/Cas9-mediated knockout of *Gbst* does not impact on reproduction ability. (**A**) Comparison of WT and *Gbst*^−/−^ 4-day total egg production. The results are shown as the mean ± standard deviation (SD). Student’s *t*-test was used to compare the differences from WT (ns: not significant, *p* = 0.74). (**B**) Comparison of WT and *Gbst*^−/−^ egg embryonic viability. The results are shown as the mean ± SD. Student’s *t*-test was used to compare the differences from WT (ns, *p* = 0.22).

The total number of eggs laid per female over a four-day period was 550 ± 92 in the WT and 524 ± 85 in the *Gbst*^−/−^, with no significant difference (*p* = 0.74, Figure 5A). Similarly, the hatching rate was 88.92 ± 2.85% for WT and 85.87 ± 2.23% for *Gbst*^−/−^, and showed no statistical significance (*p* = 0.21, Figure 5B).

### 3.6. CRISPR/Cas9 Knockout of Gbst Reduces Expression of Eye Pigmentation-Related Genes

**Figure 6:**
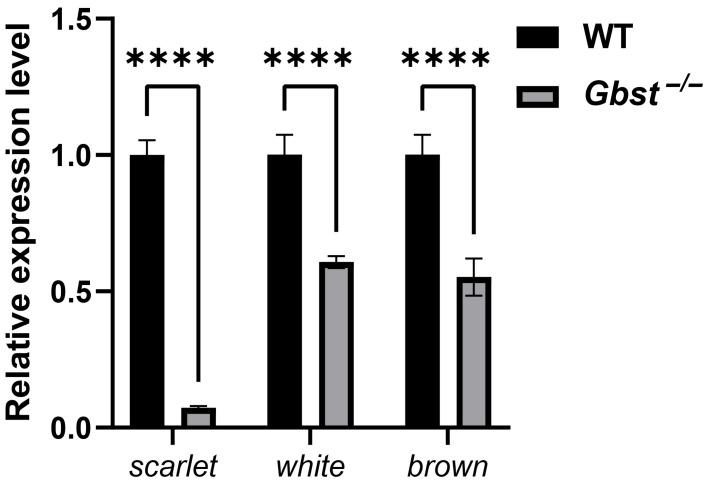
CRISPR/Cas9-mediated knockout of *Gbst* reduces expression of eye pigmentation-related genes. Relative expression levels of *scarlet*, *white*, and *brown* in WT and *Gbst*^−/−^ mutant determined by qPCR. Expression levels were normalized to the reference gene *tubulin*. Data are shown as the mean ± standard deviation (SD) from three biological replicates. Student’s *t*-test was used to compare differences from WT (**** *p* < 0.0001).

To confirm the successful knockout of *scarlet* and assess the expression profiles of other eye pigmentation-related genes, qPCR analysis was conducted for *scarlet*, *white*, and *brown*. The expression level of *scarlet* was almost undetectable in the *Gbst*^−/−^ mutant compared with the WT. In addition, the transcript levels of *white* and *brown* were significantly reduced in the mutant (**** *p* < 0.0001, Figure 6).

## 4. Discussion

The biosynthesis and transport of eye pigments are regulated by a conserved genetic network, including enzymes and ABC transporters [44]. In particular, the ABC transporter genes *scarlet*, *brown*, and *white* are known to mediate pigment transport and deposition within compound eyes. Extensive studies in model organisms, especially *D*. *melanogaster*, have provided detailed insights into the roles of these genes [29,36,45]. In this study, we cloned and characterized the *scarlet* gene in *G*. *bimaculatus,* the amino acid sequence produced from the SCARLET homolog is similar to that of other insect species, which all contain conserved 3a01204 super family domain. Phylogenetic analysis revealed high sequence similarity with orthologs in other insect species, further validating its initial annotation as a SCARLET homolog based on sequence alignment and other comparative analyses. These findings are consistent with the known role of *scarlet* in eye pigmentation reported in other insect species, suggesting that the gene identified here is a putative SCARLET homolog in *G*. *bimaculatus*. Notably, in the genome published by Ylla et al. [41], GLH03487 was annotated as a SCARLET homolog. However, our ABC transporter phylogenetic analysis demonstrates that GLH03487 does not cluster with other SCARLET orthologs (Figure S1), indicating that this sequence is not a true SCARLET homolog.

Disruption or knockout of both the ABC transporter genes and the genes encoding ommochrome biosynthetic enzymes (i.e., *vermilion*, *cinnabar*, and *cardinal*) affect pigmentation in compound eyes [44]. Furthermore, compound eye pigmentation is associated with the evolution of the insect visual system [46]. Utilizing CRISPR/Cas9 technology, the present study successfully generated a *scarlet* gene knockout in *G*. *bimaculatus*. In the *Gbst*^−/−^ knockout strain, the compound eyes displayed distinct yellow pigmentation, while the ocelli remained unchanged. The yellow-eye phenotype was detectable from the embryonic stage and persisted throughout the entire life cycle, indicating that pigment accumulation in the compound eyes begins early during development. These results suggest that *Gbst* specifically regulates pigment deposition in the compound eyes rather than in the ocelli. Similar phenotypes have been reported in hemimetabolous insects. For example, knockout of the *cinnabar* gene in *N*. *lugens* resulted in red-eye phenotypes visible in embryos, nymphs, and adults [47]. In *G*. *bimaculatus*, WT eyes are dark brown, while *white* gene mutants exhibit pure white eyes at all life stages [42]. In contrast, holometabolous insects display stage-specific pigmentation phenotypes. Knockout of the *white* gene in *H*. *armigera* affects pigment synthesis in eggs and early larvae, but eye color changes are only observed in adults [48]. Similarly, in *T*. *castaneum*, loss of the *cardinal* gene leads to eye color alterations beginning at the pupal stage [49]. These differences reflect distinct developmental strategies between hemimetabolous and holometabolous insects. In holometabolous species such as *Drosophila*, visual system development occurs in two distinct phases [46,50]. Ocelli form during embryogenesis, whereas compound eyes progressively mature through postembryonic development via successive larval molts and pupal metamorphosis. Conversely, in hemimetabolous insects like grasshoppers and crickets, eye development is a continuous process beginning in embryogenesis and extending through postembryonic stages. The developing compound eyes gradually mature and become the adult compound eyes upon completion of nymphal development [46]. These observations highlight significant differences in developmental patterns and gene regulatory mechanisms associated with distinct modes of insect metamorphosis.

Beyond its known role in pigmentation, whether *scarlet* is required for the structural integrity of compound eyes remained untested in *G*. *bimaculatus*—a gap addressed by our histological examination. Previous studies have shown that knockdown of eye determination genes, such as *sine oculis* and *eyes absent*, leads to severe defects in compound eye structure and also affects eye pigmentation [51]. In contrast, our results show that *Gbst* knockout individuals retained the typical layered architecture of the ommatidia, including the corneal lens, crystalline cone, pigment cells, rhabdom, and basement membrane. This finding indicates that *scarlet* primarily affects pigment deposition rather than the fundamental formation of compound eye structures. This difference may reflect an evolutionary divergence in the genetic regulation of eye development and pigmentation among insects, suggesting that *scarlet* homologs have conserved pigment transport roles, whereas eye determination genes influence pigmentation indirectly by affecting eye morphogenesis.

The transcript level of *scarlet* was almost undetectable in *Gbst*^−/−^ mutant, confirming the successful knockout at the transcriptional level. In addition, the expression levels of *white* and *brown* were also significantly reduced. These genes encode ABC transporter proteins that cooperate in the transport of pigment precursors in the ommochrome and pteridine pathways [29,31]. The simultaneous downregulation of *white* and *brown* implies possible transcriptional cross-regulation or functional interdependence among these transporters. Previous studies in *Drosophila* have shown that *white* and *scarlet* form heterodimeric complexes that mediate pigment precursor transport, and disruption of one component can destabilize or reduce expression of the others [31]. Therefore, the reduced expression of *white* and *brown* observed in *Gbst*^−/−^ mutant of *G. bimaculatus* may reflect similar regulatory interactions within the eye pigmentation network.

The use of eye pigmentation genes as visible markers has greatly facilitated germline transformation in various insect species, such as *D*. *melanogaster* and *Aedes aegypti* [3,47,52,53]. An ideal marker gene should allow for easy identification without requiring specialized equipment like fluorescence microscopes, and its mutation should not impair development or reproduction [54]. While some genes meet the visibility criterion, mutations lead to developmental or reproductive defects, limiting their applicability as markers. For instance, *eyeless*, involved in eye development in *Daphnia magna*, was found to be lethal when mutated [55]. Similarly, knockout of the *white* gene was embryonically lethal in *Helicoverpa armigera* [48], and caused lethality in homozygous F_1_ offspring in *Oncopeltus fasciatus* [56]. These cases highlight the importance of assessing not only visibility but also the developmental and reproductive viability of candidate marker genes. In this study, we evaluated the reproductive capacity of *G*. *bimaculatus* individuals carrying mutations in the *scarlet* gene. The results showed that *Gbst*^−/−^ mutations had no effects on egg production or embryonic viability, indicating that *scarlet* represents a suitable and visible marker gene for future transgenic applications in *G*. *bimaculatus*. In previous piggyBac- and TALEN-based transformation systems [57,58], identification of successful transformants relied on fluorescent or antibiotic markers, which required specialized equipment or additional selection steps. In contrast, *Gbst* knockout lines provide a simple visual marker system—loss of eye pigmentation can directly indicate transformation success—thereby improving the efficiency and accessibility of future genetic modification in *G*. *bimaculatus*.

## Figures and Tables

**Table 1:** Primers used in this study.

Primer Name	Sequence (5′-3′)	Purpose
*Gbst*-sgRNA-F	AATACGACTCACTATACCCGCCTGCTTC TAAGTATGT	sgRNA synthesis
*Gbst*-sgRNA-R	AAAAAAAGCACCGACTCGGTGCCAC	
*Gbst*-F	CCCGCCTGCTTCTAAGTATGT	Mutant detection
*Gbst*-R	GCTCACCCTGCGAAGAAGT	
q*scarlet*-F	TGCTCTGCACCATCCACCAG	RT-qPCR
q*scarlet*-R	GTGCCCTGCGAAGAAGTCGA	
q*white*-F	CTCATGGCTGAAGGTCGTGT	
q*white*-R	CTCTCGAGTGGGTACTACAGC	
q*brown*-F	GTGGATGCCCTGTTTTATCG	
q*brown*-R	CCACAGGCACAGATATCACA	
q*tubulin*-F	TGGACTCCGTCCGGTCAGGC	
q*tubulin*-R	TCGCAGCTCTCGGCCTCCTT	

## Data Availability

The original contributions presented in this study are included in the article/Supplementary Material. Further inquiries can be directed to the corresponding author.

## Supplementary Materials

The following supporting information can be downloaded at: https://www.mdpi.com/article/10.3390/insects17010033/s1.
