# Supplementary material for: Functional Analysis of the Scarlet Gene in the Cricket Gryllus bimaculatus
Source: Insects. 2025 Dec 25;17(1):33. doi: 10.3390/insects17010033 (PMC12842043; doi:10.3390/insects17010033)
Supplement: Supplementary file 1 [file insects-17-00033-s001.zip › insects-3875195-supplementary.pdf]

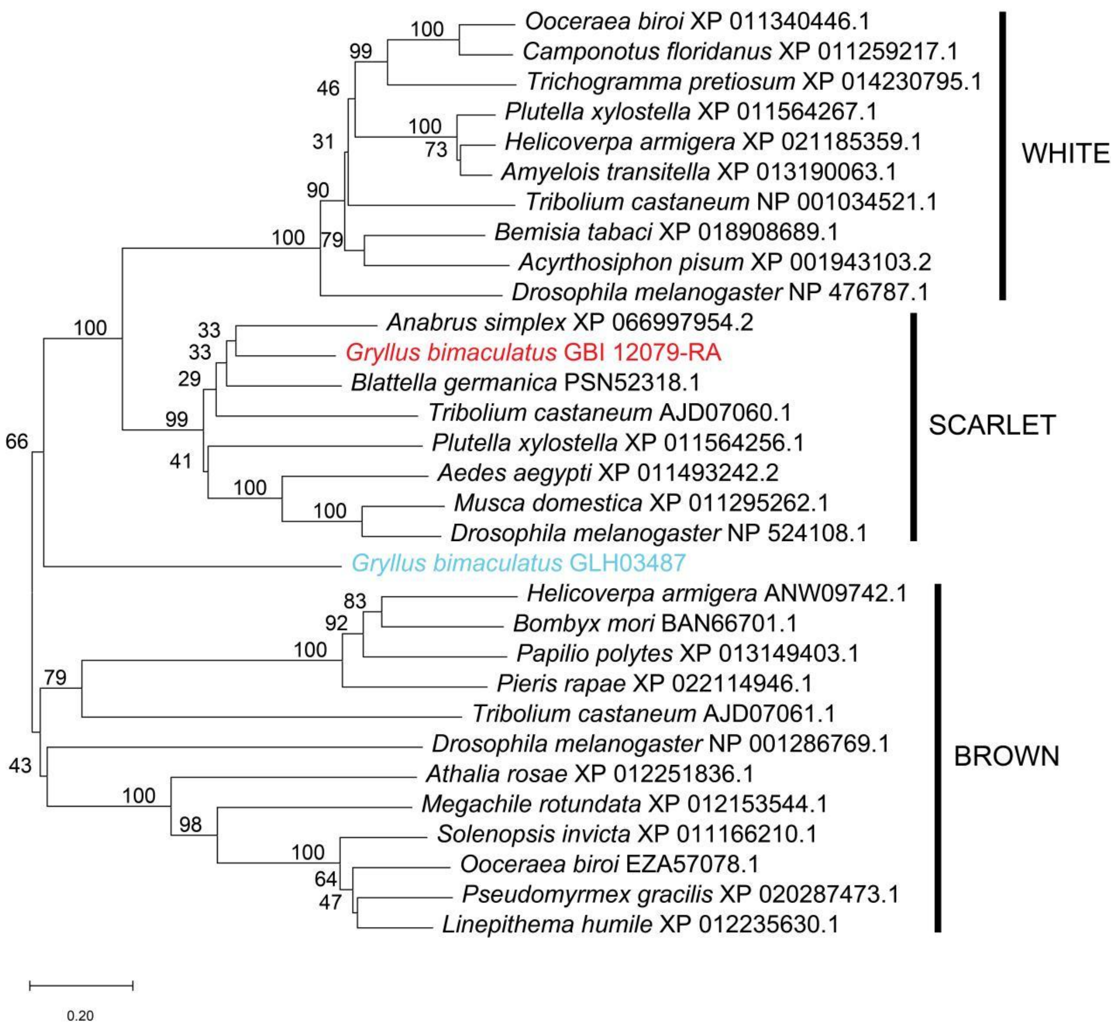

Supplementary Figure 1. Phylogenetic tree of the ABC transporter amino acid sequences among several insects. Phylogenetic tree of known ABC transporter amino acid sequences constructed by the neighbor-joining (NJ) method. GLH03487 amino acid sequence does not cluster with the canonical SCARLET amino acid sequences from other insects.
